# Pre–post intervention exploring cognitive function and relationships with weight loss, intervention adherence and dropout

**DOI:** 10.1080/21642850.2022.2162528

**Published:** 2023-01-06

**Authors:** Amanda N. Szabo-Reed, Laura E. Martin, Cary R. Savage, Richard A. Washburn, Joseph E. Donnelly

**Affiliations:** a Department of Internal Medicine, The University of Kansas Medical Center, Kansas, KS, USA; b Department of Population Health, University of Kansas Medical Center, Kansas, KS, USA; c Center for Brain, Biology and Behavior, University of Nebraska-Lincoln, Lincoln, NE, USA

**Keywords:** Obesity, weight loss, cognitive function, adherence

## Abstract

**Objective:**

To evaluate the association between baseline cognitive function, intervention dropout, adherence and 3-month weight loss (WL) when controlling for confounding demographic variables.

**Methods:**

107 (*Mage *= 40.9 yrs.), BMI in the overweight and obese range (*BMI *= 35.6 kg/m^2^), men (N = 17) and women (N = 90) completed a 3-month WL intervention. Participants attended weekly behavioral sessions, comply with a reduced calorie diet, and complete 100 min of physical activity (PA)/wk. Cognitive function tasks at baseline included Flanker (attention), Stroop (executive control) and working memory, demographics, body weight and cardiovascular fitness were assessed at baseline. Session attendance, adherence to PA and diet were recorded weekly.

**Results:**

Baseline attention was positively correlated with age (*p* < .05), education (*p* < .05), attendance (*p* < .05), diet (*p* < .05) and PA (*p* < .05). Baseline executive control (*p* < .05) and working memory (*p* < .05) were each associated with % WL. Baseline executive control (*p* < .01) and working memory (*p* < .001) were also each associated with education. ANOVA indicated that baseline attention (*p *< .01) was associated with WL, specifically for comparing those who achieved 5–10% WL (*p* < .01) and those who achieved greater than 10% WL (*p* < .01) to those who dropped.

**Significance:**

Results suggest that stronger baseline attention is associated with completion of a 3-mo. WL intervention. Executive control and working memory are associated with amount of WL achieved.

**NCT registration:**

US NIH Clinical Trials, NCT01664715

## 1. Introduction

**Figure 1.**
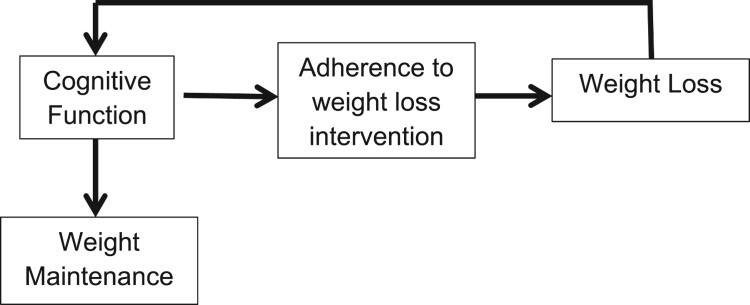
Theoretical model of the association between obesity and cognitive function.

A theoretical model of the association of obesity and cognitive function has been previously proposed by Sellbom and Gunstad (2012) (See Figure 1). Empirical support for some aspects of this model is available. For example, obesity has been associated with impaired executive control in otherwise healthy adults (Sellbom & Gunstad, 2012). Obesity and increased adiposity earlier in life may be linked to both an increased incidence of Alzheimer’s disease in older individuals, and cognitive deficits in younger adults prior to the onset of dementia (Gunstad, 2007, 2010). Cross-sectional data from over 400 adult participants in the Baltimore Longitudinal Study (BLS) suggested that memory performance was impaired in obese individuals across the adult lifespan (age 21–82 yrs.) as well as in younger and middle-aged adults (age 21–50 yrs.) (Gunstad, 2007, 2010). Among BLS participants, higher baseline waist circumference, an indicator of central obesity, was associated with reduced performance on global cognitive function, memory, and language tasks at baseline and more rapid declines in global cognitive functioning, executive function, and memory over time (Gunstad, 2010).

### 1.1. Cognitive function and adherence to behavior change

Adoption and maintenance of health behavior change requires high levels of cognitive function often in the form of executive control which is centered around engaging in goal directed problem solving, inhibition of habitual unhealthy behaviors, and self-control. Although executive control has been associated with adherence to diet (Fieldhouse, 2020; Szabo-Reed, 2020) and smoking interventions (Masiero et al., 2020), as well as interventions to treat addictions (Domínguez-Salas, 2016), limited information is available regarding the role of cognitive function, including executive control, in adherence to a traditional weight loss intervention using energy restriction and increased exercise. Spitznagel (2013) reported an association between baseline cognitive performance and weight loss 12 months following bariatric surgery, indirectly suggesting that better cognitive performance was associated with better adherence to bariatric postoperative guidelines. Results from the exercise literature suggest that higher baseline executive control maybe associated with improved adherence to an exercise intervention (McAuley, 2011). A sample of 177 older adults (mean age 66.4 yrs.) were randomly assigned to either a walking or a flexibility/toning/balance group which met 3 days/wk. over 12 months. Attendance over the last 11 months. of the trial represented adherence. Structural equation modeling results suggested that higher executive control and the use of self-regulatory strategies enhanced an individual’s belief in their personal exercise capabilities, which in turn, resulted in improved exercise adherence.

### 1.2. Weight loss and cognitive function

There is evidence to suggest cognitive function predicts weight loss intervention adherence and that intentional weight loss is associated with improved executive control. For example, Butryn (2019) enrolled 309 adults into a 6 month behavioral weight loss program emphasizing self-monitoring of calorie intake and 250 min of moderate-to-vigorous physical activity per week. Executive function was measured pre and post intervention using the Delis-Kaplan Executive Function System (D-KEFS). Baseline D-KEFS achievement score, rule violations, and completion time significantly predicted weight loss at 6 months. Rule violations also significantly predicted physical activity at 6 months. These findings suggest that aspects of executive functioning may predict eating and exercise-related behaviors. Similarly, Szabo-Reed (2020) found that brain activation in areas of the brain associated with executive control (i.e. middle frontal gyus and dorsolateral prefrontal gyrus) in *N* = 75 adults with overweight or obesity was related to diet, physical activity and subsequent weight loss during a 3 month weight management intervention modifying both diet and exercise behaviors. Together these studies suggest that baseline cognitive function, and associated brain function, may be predictive of weight management intervention adherence and subsequent weight loss.

Several studies have noted improve cognitive functioning is associated with intentional weight loss. Brinkworth (2009) reported improved working memory and processing speed in a sample of overweight and obese individuals (BMI = 33.7, age = 50 yrs.) who lost and average of 13.7 kg following either a low-carbohydrate/high fat or high carbohydrate/low fat diet over 1 yr. Smith (2010) showed that participants on the DASH diet combined with a behavioral weight management program exhibited greater improvements in executive function-memory-learning and psychomotor speed, and DASH diet alone participants exhibited better psychomotor speed compared with a usual diet control. Neurocognitive improvements appeared to be mediated by increased cardiovascular fitness and weight loss. Siervo (2012) reported a 9.7% weight loss in a sample of middle aged (30–59 yrs.) and older (≥60 yrs.) obese (BMI 30–50) was associated with improved cognitive performance as assessed by the Trail Making Test. However, this result is potentially biased as only 42% of the baseline sample completed weight loss. Weight loss induced by bariatric surgery has also been show to improve attention, executive function and memory (Alosco et al., 2014). Recently, Gowey and colleagues (2021) found that individuals with obesity who achieved clinically significant weight loss via a behavioral intervention have average to above average executive function. The authors also noted that individuals who maintained their weight loss for at least one year, as compared to those who regained, performed better on tests of decision making. Finally, Peven et al. (2020) enrolled 125 middle-aged adults with overweight and obesity into a 12-month behavioral weight loss intervention. Participants were assigned to one of three groups: energy-restricted diet alone, an energy-restricted diet plus 150 min of moderate intensity exercise per week or an energy restricted diet plus 250 min of exercise per week. All participants completed tests measuring executive functioning and/or reward sensitivity, including the Iowa Gambling Task (IGT). Following the intervention, weight significantly decreased in all groups. The authors found a significant multivariate effect of group on cognitive changes and a Group × Time interaction only on IGT reward sensitivity, such that the high exercise group improved their performance relative to the other two intervention groups. There was also a main effect of Time, independent of intervention group, on IGT net payoff score. Changes in weight were not associated with other changes in cognitive performance. Overall, the authors concluded that, engaging in a high amount of exercise improved reward sensitivity above and beyond weight loss alone. This suggests that there is additional benefit to adding exercise into behavioral weight loss regimens on executive functioning, even without additional benefit to weight loss.

### 1.3. Confounders and cognitive function

Important potential confounders have often been shown to be associated with cognitive function and weight. For example, a large body of literature suggests that cardiovascular fitness has a positive association with cognitive function in older adults (Colcombe, 2006; Kramer, 2005; Kramer et al., 2006). In addition, weight loss in programs including a physical activity component are associated with an increase in cardiorespiratory fitness (Aadland & Robertson, 2012). However, there is a lack of studies valuating the association between cardiorespiratory fitness, weight, and cognition in middle aged adults. Similarly, IQ has consistently been shown to be associated with commonly used neuropsychological assessments evaluating cognitive function (Starr, 1992) and is also linked to cognitive aging which is negatively impacted by increased weight (Gunstad, 2007, 2010; Stern, 2012). Education level has is also associated with cognitive function outcomes across the life span (Lövdén, 2020). Unfortunately, these important confounding variables are often not assessed or included in weight management trials.

Current evidence suggests that higher levels of cognitive function may be associated with greater adherence to a weight loss intervention and that weight loss may be associated with improved cognitive function. However, the current literature does not often control for potential confounders, most importantly cardiovascular fitness, IQ or education level or include multiple assessments of cognitive function. To reduce these gaps in the current literature, we aimed to investigate the influence of baseline cognitive function on 3-month weight loss and program adherence for individuals with a body mass index (BMI) in the overweight and obese range participating in a structured weight management program (Szabo, 2013; Washburn, 2021) when controlling for potential confounders. We hypothesize that when controlling for important potential confounders, baseline cognitive function will be associated with subsequent weight loss at 3 months and study drop out.

## 2. Methods

A detailed description of the design and methods for this trial has been published (Szabo, 2013). Briefly, participants (*n* = 287) were recruited from Fall of 2012 to Fall of 2015 from Kansas City and the surrounding areas to complete a 3-mo. weight loss intervention of decreased energy intake (1200–1500 kcal/d) and increased exercise (100 min/week). Outcome assessments were completed prior to weight loss (−3 months) and at completion of weight loss (0 months). A fasting baseline weight was collected first followed by cardiorespiratory fitness and finally IQ and cognitive function. Each outcome was collected at a separate scheduled appointment. The primary outcomes for this trial can be found here (Washburn, 2021). Aspects of the Prevention of Weight Regain (POWeR) trial relevant to the current proposal are described in detail in the following sections.

### 2.1. Participants

A subset of participants enrolled in the parent trial (Washburn, 2021) *N* = 107 (*Mage *= 40.9 yrs.) healthy, sedentary, individuals with a body mass index (BMI) in the overweight and obese range (*BMI *= 35.6 kg/m^2^), adult men (*N* = 17) and women (*N* = 90) who were able to exercise and willing to be randomized into one of three groups (i.e. 150, 225 or 300 min of exercise during the 12 month weight maintenance period) completed a 3 month weight loss intervention. Participants also needed clearance from their primary care physician to participate. Participants were excluded if they participated in a research project involving weight loss or exercise in the previous 6 months, currently participating in a regular exercise program, i.e. >500 kcal/week. of planned activity assessed by questionnaire (20), not weight stable (>4.5 kg) for 3 months prior to intake; pregnant during the previous 6 months, lactating, or planned pregnancy in the following 15 months; serious medical risk such as type 1 diabetes, cancer or recent cardiac event, i.e. heart attack, angioplasty, etc.; eating disorder, current treatment for psychological issues or taking psychotropic medications; taking medications known to affect weight; adherence to specialized diets and no access to grocery shopping and meal preparation, i.e. military, college students with cafeteria plan, etc.

### 2.2. Weight loss intervention

Weekly 60 min in-person behaviorally based clinics were conducted during weight loss. All clinics used behavioral strategies based on Social Cognitive Theory to promote change in both diet and exercise (Bandura, 1986, 2004). Energy intake was reduced to ∼1200–1500 kcal/day using a combination of commercially available portion-controlled entrees, fruits and vegetables, low calorie shakes, and non-caloric beverages. Participants were provided with portion-controlled entrees, fruits and vegetables, shakes, and non-caloric beverages. Participants consumed a daily minimum of 2 entrees (180–270 kcals each), at least 5 servings of fruits and/or vegetables, and 3 shakes (∼100 kcal each). Non-caloric beverages such as diet soda, coffee, etc. were also allowed ad libitum. Exercise during weight loss progressed from 10 min/d, 5 d/wk, 65% age-predicted maximal heart rate (HR_max_ = 220 – age in yrs.) to the goal of 20 min/d, 5 d/wk, 70% HR_max_ (100 min/wk) at week 7 and remain at this level through study week 12. The intensity and duration of both on-site supervised (minimum 3 sessions/wk) and non-supervised exercise (walking or running) sessions were verified by a HR monitor.

### 2.3. Outcome assessments

*Cognitive function*: Following recommendations of Miyake (2000) and Salthouse (2005) we used multiple measures of cognitive function. These measures of cognitive function have been associated with adherence to an exercise intervention (McAuley, 2011). These include Flanker task, Stoop color-word task, and two working memory tasks from the Virginia Cognitive Aging Project, spatial relations, and matrix reasoning. Each participant completed his/her cognitive tasks in a randomized order. *Flanker task*: The modified flanker paradigm (Botvinick, 1999; Voss et al., 2010) required participants to identify the orientation of a central arrow cue that is flanked by arrows that are in either a congruent (e.g. >>>>>) or incongruent (e.g. >><>>) orientation. This task assesses attention or the ability to choose and concentrate on relevant stimuli (Lamers & Roelofs, 2011). The outcome of interest is cost or inhibition and was calculated by subtracting the reaction times of congruent trials from those of incongruent trials and dividing by the reaction time of congruent trials. This approach accounted for individual differences in perceptual speed. *Stroop color-word task*: The modified Stroop task (Prakash, 2009) consisted of four trial types: congruent, neutral, incongruent–eligible, and incongruent–ineligible. This task is a measure of cognitive flexibility that encompasses the selection and processing of task-relevant over task-irrelevant information, thereby enabling goal-directed behavior even in the face of habitual but incorrect response tendencies. This type of task also enables individuals to flexibly adjust their response to changing contextual demands (e.g. increasing focus on task-relevant information or strongly filtering irrelevant information when likelihood of distraction is high but relaxing control when it is low) (Braver, 2012; Cohen-Shikora et al., 2018). For this measure of cognitive flexibility, the congruent condition consisted of words presented on a screen that are printed in an ink color that matched the color of the stimuli (e.g. BLUE in blue color), whereas the neutral condition consisted of words that were matched for the length and frequency of the color of the stimuli but not color category (e.g. SHIP in blue color). The incongruent–eligible stimuli involved the presentation of words that matched one of the potential responses (e.g. BLUE in red ink if ‘blue’ was one of the potential responses). Incongruent–ineligible stimuli presented words that did not match the set of potential responses (e.g. PURPLE in blue ink if ‘purple’ is not one of the potential responses). Two ink-color sets were used and counterbalanced across participants who were instructed to respond as quickly and as accurately as possible. The outcome measure was calculated as the mean Reaction Time for the Incongruent trials minus Reaction Time for congruent trials. This provides a measure of cost interference whereby higher scores represent less interference. The spatial relations task and the matrix reasoning are both part of the Virginia Cognitive Aging Project (Salthouse, 1989, 1993, 2005). *Spatial relations task*: Participants are asked to determine which three-dimensional figure can be formed from a presented two-dimensional pattern. This task measures visuoconstruction ability and working memory by requiring participants to remember information while also carrying our specified processing (Salthouse, 1989). The outcome for this task is the total number of items responded correctly to Salthouse (1989). *Matrix reasoning*: During this computer task, participants were asked to determine which pattern best fits the missing cell that completed the presented matrix. The outcome for this task was total number of items responded to correctly. This task measures visuospatial ability and working memory by similarly requiring participants to remember information while also visuospatially manipulating it (Raven, 1989; Salthouse, 1993).

*Education* the number of years of schooling completed (i.e. 12 = high school, 14 = associates degree, 16 = bachelor’s degree, etc.).

*Estimated Intelligence quotient (IQ)* was assessed using the Wechsler Abbreviated Scale of Intelligence to provide a brief, reliable measure of cognitive ability based on vocabulary and matrix reasoning (Goldstein & Mazefsky, 2013).

*Body weight* was obtained using a digital scale accurate to ±0.1 pounds (Befour Inc. Model #PS6600, Saukville, WI). Participants reported to the laboratory between 6 and 10 AM, after an overnight fast, and were weighed wearing a standard hospital gown prior to breakfast and after attempting to void.

*Cardiovascular fitness* was assessed using a sub-maximal treadmill test. The treadmill speed was set a 3 mph, 0% grade. Speed remained constant and grade increased 1% each minute until the participant reached either 75% age predicted HR max (participants not on beta blockers) or a rating of perceived exertion (RPE) of 16 on the Borg Scale (Borg, 1982) (participants on beta blockers). HR was obtained by electrocardiogram (Marquette Electronics, Milwaukee, WI, U.S.A.) and perceived exertion was assessed during the last 15 s of each stage. Cardiovascular fitness was defined as the metabolic equivalent (MET) level estimated from treadmill speed and grade achieved during the last stage completed after achieving either the HR or RPE criteria.

*Intervention adherence*: Health educators conducting behavioral clinics tracked attendance to clinic meetings, the number of portion-controlled entrees consumed, fruit and vegetable intake and eating off plan. Adherence to clinic meetings was determined based upon total number of sessions attended divided by the number of sessions available. Adherence to portion-controlled meals, fruit and vegetable intake, and eating off plan was determined based on the number consumed divided by the number recommended for each item. The frequency and duration of both supervised and unsupervised exercise sessions was documented by each participant on an exercise log. Supervised exercise logs were also maintained by staff. Each participant was prescribed 100 min of physical activity to complete each week.

### 2.4. Data analysis

As previously indicated only the baseline (0) and post-weight loss (-3 months) outcome assessments were utilized for this analysis. We examined each measure of cognitive function independently as previous research suggests that each measure is truly unique (Miyake, 2000). Data analysis was performed in SPSS 27 (Corp., 2020). Initial Pearson correlations were conducted to determine the relationship between each measure of cognitive function, weight, cardiovascular fitness, and intervention adherence (Supplemental Table 1 contains the correlations between the cognitive function tasks). Hierarchical linear regression was then used to evaluate the association of each measure of baseline cognitive function with measures of adherence to both the diet energy intake at mo. 0, average consumption of portion-controlled entrees, shakes, fruits/vegetables, and number of clinic sessions attended (−3 to 0 months) and exercise (number of prescribed exercise sessions successful completed (−3 to 0 months). We also used multiple linear regression to evaluate the association of each measure of baseline executive control and percent weight loss (−3 to 0 months). Analysis of variance (ANOVA) was used to determine the relationship between weight loss, intervention drop out and cognitive performance. Kruskal–Wallis results are reported when inequal groups are present. All missing data was left and reported as missing.

### 2.5. Ethics statement

This study was approved by the Human Subjects Committee at the University of Kansas Medical Center (IRB 19,775).

## 3. Results

One hundred and seven individuals, a subset of those who initially began the weight loss phase for the POWeR study (Washburn, 2021), completed the cognitive assessments at baseline. The sample had a mean age of 40.92 (8.07) year, was 84.1% female and contained 39.2% racial/ethnic minorities (See Table 1). The mean weight loss over the 3-month weight loss period was 8.70% (±3.34%). Categorically, 9.3% of the sample lost less than 5% of their baseline body weight, 46.7% lost 5–10%, 29.9% lost more than 10% and 14% dropped from the study during the first 3 months (See Table 2).

**Table 1.** Sample descriptive.

	Mean (SD) OR %	Min	Max	% Missing
Age	40.92 (8.07)	23	55	0%
Female	84.1%			0%
% Minority	54.2%			0%
Years of education	15.59	10	23	6.5%
IQ (Wasi percentile)	63.88 (27.25)	7	99	2%
Baseline weight (LBS)	219.77 (36.63)	146.60	319.85	0%
Weight change (LBS)	−19.29 (8.59)	−47.42	−4.70	12.1%
% Weight change	−8.70% (3.34%)	−18.93%	−2.46%	12.1%
% Clinic sessions attended	84.69% (21%)	7.69%	100%	0%
% Shakes consumed	74.94% (23.74%)	7.04%	101.11%	0.9%
% Entrees consumed	76.98% (23.04%)	7.22%	108.89%	0.9%
% Fruits and vegetables consumed	89.59% (31.38%)	4.89%	207.33%	0.9%
% Physical activity minutes	88.81% (34.73%)	1.30%	174.78%	0.9%
# Of off diet episodes	15.33 (11.66)	0	55	0.9%

**Table 2.** Weight loss category.

	%
Less than 5%	9.3%
5–10%	46.7%
Greater than 10%	29.9%
Drop	14%

Correlations (See Table 3) showed that Flanker performance was associated with age (*r*(104) = .196, *p* = .047), years of education (*r*(97) = .215, *p* = .034), percent of clinic sessions attended (*r*(104) = .235, *p* = .016), percent of shakes consumed (*r*(103) = .242, *p* = .014), percent of entrees consumed (*r*(103) = .276, *p* = .005), percent of total recommended fruits and vegetables consumed (*r*(103) = .345, *p* = .000) and percent of total physical activity minutes completed (*r*(103) = .224, *p* = .023). This suggests that better performance on the flanker task at baseline was independently associated with being younger, more years of education, attended more clinic sessions, consumed more shakes, entrees, fruit and vegetables, and completed more minutes of physical activity during the 3-month weight loss phase. Similarly, Stroop (*r*(91) = .214, *p* = .042), spatial relations (*r*(91) = −.251, *p* = .017), and matrix reasoning (*r*(91) = −.236, *p* = .025) were each associated with percent weight change. This suggests that individuals who performed better on the Stroop, spatial relations and matrix reasoning lost more weight over the course of the 3 months. Stroop (*r*(97) = .300, *p* = .003), spatial relations (*r*(98) = −.351, *p* = .000), and matrix reasoning (*r*(98) = −.413, *p* = .000) were each also associated with years of education. As expected, more years of education was positively associated with performance. Similarly, IQ was associated with spatial relations (*r*(104) = .302, *p* = .002) and matrix reasoning (*r*(104) = .495, *p* = .000), as expected higher IQ score was associated with better performance. Other potential covariates including baseline fitness were not associated with any of the measures and therefore removed from future analyses. Given that IQ was highly correlated matrix reasoning, we decided to remove it from future analysis due to collinearity as the WASI contains a matrix reasoning task.

**Table 3.** Correlation coefficients.

	Age	ED	IQ	% Weight change	% Of classes attended	% Shakes consumed	% Entrees consumed	% Fruits and vegetables consumed	% Physical activity minutes	# Of off diet episodes
Flanker	.196*	.215*	−.003	−.129	.235*	.242*	.276**	.345**	.224*	.108
Stroop	−.175	.300**	.129	−.214*	.019	−.048	−.047	−.021	.009	.136
Spatial relations	−.188	.351**	.302**	−.251*	−.010	.038	−.001	.083	.014	.168
Matrix reasoning	−.108	.413**	.495**	−.236*	−.003	.090	.040	.168	.032	.167

* *p* < .05.

**p < .01.

Multiple linear regression showed a lack of significance of flanker performance, years of education and age on percent weight change *F*(3,83) = 0.865, *p* = .463, *R*^2 ^= .031. We also fit a model adjusted for Stroop performance, years of education and age on percent weight change (*F*(3,83) = 2.029, *p* = .116, *R*^2 ^= .071) and again found a lack of significance. A model fit for spatial relations, years of education and age on percent weight change (*F*(3,83) = 2.289, *p* = .085, *R*^2 ^= .079). Finally, we fit a model adjusted for matrix reasoning, years of education and age on percent weight change (*F*(3,83) = 1.659, *p* = .182, *R*^2 ^= .059). These findings suggest that cognitive performance at baseline is not associated with percent weight change when controlling for confounders such as years of education and age.

To examine the relationship between baseline cognitive performance, subsequent weight loss and drop out, participants were divided into four subgroups (See Table 2). Results of an ANOVA indicated that there was a difference in flanker performance between weight loss categories (*F*(5, 93) = 4.171, *p* = .008, partial *η*^2^ = .119) when adjusting for years of education and age. Post-hoc t-tests indicated that the difference was between those who achieved 5-10% weight loss and those who dropped (*t* = .065, *p* = .009) and those who achieved greater than 10% weight loss and those who dropped (*t* = .067, *p* = .012). Kruskal–Wallis Test. *H*(3) = 9.942, *p* = 0.019 and confirmed flanker performance was significantly different by weight loss group despite inequal group sizes. There was not a difference in Stroop performance between categories (*F*(3, 92) = 0.666, *p* = .575, partial *η*^2^ = .021) when adjusting for years of education. There was not a difference in spatial relations between weight loss categories (*F*(3, 93) =  1.393, *p* = .250, partial *η*^2^ = .043) when adjusting for years of education. There was also a no significant difference in matrix reasoning between weight loss categories (*F*(3, 93) =  .948, *p* = .421, partial *η*^2^ =  .030) when adjusting for years of education. These findings suggest that those who performed better on the flanker task achieved greater weight loss at 3 months and were less likely to drop out of the weight management program when controlling for years of education. A chi square analysis conducted to evaluate the proportion of White as compared to racial/ethnic minorities in each weight loss subcategory is present in Table 4. Although not significant, the chi square results suggest that individuals who report being an ethnic/racial minority achieved less weight loss at 3 months and were more likely to drop. However, a larger sample is needed to statistically interpret these findings.

**Table 4.** % Within weight loss category by race/ethnicity.

	% White	% Minority
Less than 5%	40%	60%
5–10%	62%	38%
Greater than 10%	53.1%	46.9%
Drop	40%	60%

## 4. Discussion

Overall, results indicated that attention (flanker task) was positively correlated with session attendance, adherence to the diet and physical activity. Baseline attention was negatively associated with dropout and suggested that those with high attention prior to starting a weight management intervention, achieved greater weight loss and were less likely to dropout. Finally, although not significant, the chi square results suggest that individuals who report being an ethnic/racial minority achieved less weight loss at 3 months and were more likely to drop from the study.

Attention is an important aspect of cognitive functioning (Diamond, 2013). However, few studies have explored the association between attention and adherence to weight management programs. In the present study we found attention was not only related to weight loss at 3 months, but poor baseline attention was associated with program dropout. A recent meta-analysis found that weight loss was associated with a significant improvement in attention and memory in both longitudinal studies and randomized control trials (RCTs), whereas executive function and language improved in longitudinal and RCT studies, respectively (Veronese, 2017). Similarly, weight loss induced by bariatric surgery has also been show to improve attention, executive function and memory (Alosco et al., 2014; Masiero et al., 2020), measured executive function using the D-KEFS which captures planning, inhibitory control, and mental flexibility. This group found that baseline executive function was predictive of weight loss at 6 months. Additional research is needed to explore the association between attention and adherence to weight management.

Like other studies, we found that the influence of cognitive function on weight management may be domain specific or associated with a specific type of cognitive function rather than global cognition. Gowey (2021) found that executive function, specifically decision-making performance was better in those who maintained weight loss as compared to those who regained. They hypothesize that this association may be related to food decision-making specifically, fruit/vegetable intake (Higgs & Spetter, 2018). However, they did not evaluate this. Similar to Butryn (2019) and Galioto (2016) we found executive function (i.e. Stroop performance) at baseline was associated with weight loss. The present investigation also found that baseline attention was associated with better clinic session attendance, diet consumption (i.e. shakes, entrees, fruits/vegetables) and physical activity, while executive function, specifically cognitive flexibility and working memory, were associated with greater weight loss at 3 months. This supports Gowey (2021) speculation that specific cognitive domains may be related to behaviors that are associated with successful weight loss. However, additional research is needed to conclude which cognitive domains are associated with successful behavior changes linked to weight loss success (Szabo-Reed, 2015).

IQ, years of education and performance on measures of cognition are often highly related (Ganuthula & Sinha, 2019). This statement rings true for the findings observed in the present study. We found that attention, executive control and working memory were correlated with weight loss, however when years of education controlled for in the analysis the association was minimized. Previous research in this arena has failed to measure IQ or years of education (Butryn, 2019; Galioto, 2016). However, other covariates including race/ethnicity have been evaluated. Butryn (2019), assessed the influence of race on executive function and weight loss. They found that when race was added as a covariate to the model, this prediction became nonsignificant. To explore the association between of race/ethnicity and weight loss we conducted an exploratory chi-square analysis and found that minority status was associated less weight loss at 3 months and higher rates of dropout. However, no significance was found. Even so, additional research is needed to understand the complex relationships between cognition, behavior change, education, and race.

The present study, combined with previous research, provides promising evidence that cognitive function is associated with not only weight loss outcomes, but also potentially adherence to weight loss behaviors. Specifically, we found that baseline attention was associated with better clinic session attendance, diet consumption (i.e. shakes, entrees, fruits/vegetables) and physical activity, while executive function (i.e. cognitive flexibility, working memory) were associated with greater weight loss at 3 months. Johnston and colleagues (Johnston, 2019) recently found that during a 6-month online weight loss intervention, increased likelihood of achieving a weight loss ≥5% to <10% was associated with attending approximately one-third (35.4%) of weekly meetings, use of the member website about 25% of days, and use of the mobile application 16.1% of days. Attendance at approximately two-thirds (64.5%) of meetings, use of the member website 41.6% of days, and use of the mobile application 14.7% of days were associated with a clinically significant weight loss of ≥10%. Meeting attendance was the strongest predictor of weight loss at 6 months. Similarly, Szabo-Reed (2015) found attendance and other weight loss behaviors (diet and physical activity) we associated with weight loss success. However, both studies did not study how cognitive function as associated with such adherence. Future studies should continue to explore this concept as there appear to be an association between not only weight loss behaviors and weight loss outcomes, but also cognition, behavior, and subsequent weight loss outcomes.

Previously, Szabo-Reed (2020) found that brain activation in the Middle Frontal Gyrus was associated with 3-month weight loss and attendance to weight management intervention clinic sessions. This study suggests that cognitive measures linked to self-regulation, which include planning and decision-making that are carried out in prefrontal cortex, are linked to health behavior adherence and change (Hall & Marteau, 2014; Miyake, 2000). The right middle frontal gyrus (BA 9), has also been linked to dietary self-control and attention to health cues (Hare et al., 2009, 2011). Activation in the right middle frontal gyrus (BA 8) has also been predictive of future weight loss (Murdaugh, 2012; Szabo-Reed, 2020). Unfortunately, imaging was not able to be completed within the present study, however, previous research suggests that future studies should continue to explore the relationship between baseline cognitive function, brain structure/function, intervention behaviors, and weight loss outcomes.

Future research should consider designing larger and powered trials to evaluate the impact of cognitive function on weight loss and weight maintenance. Although this study represents a step in that direction, even though they are statistically significant, many of the outcomes (i.e. correlations), can be considered weak. Further evaluation of demographic factors including education and race/ethnicity should also be conducted. Specifically, additional research needs to explore the impact of educationally or racially tailored interventions on adherence. In addition, research evaluating ways to improve cognitive function prior to a behavioral weight management intervention. Such interventions could include cognitive training which has been suggested as a means for increasing executive control and subsequent behavioral mechanisms that are required for weight loss success (Szabo-Reed & Donnelly, 2021). Finally, additional research between baseline cognition, weight management behaviors and outcomes should also include mechanism research. This could include the use of MRI to evaluate brain mechanisms associated with weight management outcomes.

In conclusion, the present study investigated whether baseline cognitive function may be associated with greater adherence to a weight loss intervention when adjusting for important demographic factors. We found a positive correlation of attention with session attendance, adherence to the diet and physical activity. Executive control and working memory were also positively correlated with weight loss at 3 months. We also found that attention, executive control and working memory, combined, were associated with weight loss. Baseline attention was negatively associated with dropout and suggested that those with high attention, achieved greater weight loss and were less likely to dropout. Those with better working memory at baseline also showed an overall trend with achieving higher weight loss at 3 months. Although this is an initial step in better understanding the contribution of baseline cognitive function on behavioral weight management outcomes additional research is clearly needed to better define mechanisms of action and evaluate interventions that can influence baseline executive control and subsequent behavioral outcomes and demographic factors.

## Supplementary Material

Supplemental Material

## Data Availability

The data that support the findings of this study are available from the corresponding author, ANSR or JED, upon reasonable request.

## Open Scholarship

This article has earned the Center for Open Science badge for Preregistered. The materials are openly accessible at NCT01664715
