# Supplementary material for: Pre–post intervention exploring cognitive function and relationships with weight loss, intervention adherence and dropout
Source: Health Psychol Behav Med. 2023 Jan 6;11(1):2162528. doi: 10.1080/21642850.2022.2162528 (PMC9828788; doi:10.1080/21642850.2022.2162528)
Supplement: Supplemental Material [file RHPB_A_2162528_SM4829.docx]

Supplemental Table 1. Correlation coefficients between cognitive assessments

|  | FLANKER | STROOP | SPATIAL RELATIONS | MATRIX REASONING |
| --- | --- | --- | --- | --- |
| Flanker |  | .162 | -.121 | -.061 |
| Stroop |  |  | .162 | .195* |
| Spatial Relations |  |  |  | .629** |

* p<.05

**p<.01
